# The pupil's response to affective pictures: Role of image duration, habituation, and viewing mode

**DOI:** 10.1111/psyp.12668

**Published:** 2016-05-13

**Authors:** Robert J. Snowden, Katherine R. O'Farrell, Daniel Burley, Jonathan T. Erichsen, Naomi V. Newton, Nicola S. Gray

**Affiliations:** ^1^ School of PsychologyCardiff UniversityCardiffUK; ^2^ School of Optometry and Vision SciencesCardiff UniversityCardiffUK; ^3^ School of MedicineSwansea UniversitySwanseaUK

**Keywords:** Affect, Pupil dilation, Emotion, Habituation, Attention

## Abstract

The pupil has been shown to be sensitive to the emotional content of stimuli. We examined this phenomenon by comparing fearful and neutral images carefully matched in the domains of luminance, image contrast, image color, and complexity of content. The pupil was more dilated after viewing affective pictures, and this effect was (a) shown to be independent of the presentation time of the images (from 100–3,000 ms), (b) not diminished by repeated presentations of the images, and (c) not affected by actively naming the emotion of the stimuli in comparison to passive viewing. Our results show that the emotional modulation of the pupil is present over a range of variables that typically vary from study to study (image duration, number of trials, free viewing vs. task), and encourages the use of pupillometry as a measure of emotional processing in populations where alternative techniques may not be appropriate.

When a person becomes aroused or excited, the pupil enlarges (Snowden, Thompson, & Troscianko, 2012). Given this response, it is not surprising that researchers have used pupillometry as a physiological measure of psychological processes (Laeng, Sirois, & Gredeback, 2012). In particular, the pupil has been seen as an easily accessible window to examine the processing of emotional stimuli (Bradley, Miccoli, Escrig, & Lang, 2008), and the modulation of the pupil size due to the emotional content of an image has become a paradigm in which to investigate individual differences in emotional processing (for examples, see Laeng et al., 2012). In the present paper, we examine whether certain parameters that often vary across experiments may serve to eliminate, reduce, or even enhance this effect.

The pupil of the eye is actually a virtual structure, being a hole in the middle of the contractile pigmented iris. At any given moment, the pupil controls the amount of luminance reaching the retina, but light level and the state of retinal adaptation are not the sole influence on the actual size of the pupil. Early work in this area (Hess & Polt, 1960) showed that the pupil appears to react to the specific meaning of the pictures rather than the mere presentation of patterns of light and dark. For example, pictures of male pin‐up figures produced larger pupil dilations in women, whilst men's pupils dilated more to pictures of female pin‐ups.

Pupillary movements are controlled by the iris, which is in turn controlled by the opposing components of the autonomic nervous system (Beatty & Lucero‐Wagoner, 2000). The constrictor muscle is innervated by the parasympathetic division, whereas the dilator muscle is regulated by the sympathetic division, suggesting that pupil dilation reflects sympathetic nervous system activity. The dynamic “push‐pull” balance between the level of contraction of these two muscles within the iris determines pupil diameter. Hence, dilation of the pupil occurs due to increased activity in the sympathetic system or inhibition of parasympathetic activity, and constriction of the pupil occurs as a result of increased parasympathetic activity or reduced sympathetic activity (Steinhauer, Siegle, Condray, & Pless, 2004). Hence, pupillometry is a method that might give objective insight into a person's state of emotional arousal free from the bias of self‐report.

Seminal work by Bradley et al. (2008) has indeed demonstrated that the pupil is sensitive to the emotional content of pictures. They demonstrated that the pupil was larger when a picture that had emotional content was viewed compared to one that was emotionally neutral. Further, the valence of this emotion was not important—pupil dilation occurred equally to visual stimuli depicting both positive and negative scenes. These basic findings have now been replicated on several occasions (Arriaga et al., 2015; Bradley & Lang, 2015; Geangu, Hauf, Bhardwaj, & Bentz, 2011; Henderson, Bradley, & Lang, 2014; van Steenbergen, Band, & Hommel, 2011). In the original Bradley et al. (2008) study, pupil dilation also covaried directly with skin conductance changes, suggesting that pupil diameter during emotional picture viewing reflects sympathetic nervous system activity. Therefore, it is argued that the pupil response, when other relevant factors are controlled for, indirectly represents a process that signals defensive and alerting reactions to motivationally salient stimuli (Cunningham & Brosch, 2012). Hence, changes in pupil size in response to emotional stimuli could be a highly valuable tool for investigations where levels of emotional processing need to be assessed, such as in offender or patient populations (Burkhouse, Siegle, Woody, Kudinova, & Gibb, 2015; Kuchinke, Schneider, Kotz, & Jacobs, 2011; Lemaire, Aguillon‐Hernandez, Bonnet‐Brilhault, Martineau, & El‐Hage, 2014; Nuske, Vivanti, Hudry, & Dissanayake, 2014). Pupillometry may also be valuable in populations that cannot report on their emotions (e.g., infants or nonhumans) or may not be reliable in their self‐report due to lack of insight or unwillingness (e.g., psychopaths), or where wearing electrodes is not viable (such as in fMRI scanners)—see Hepach and Westermann (in press).

## Experiment 1. The Pupil's Response to Emotions: Target Duration

Previous experiments looking at the effects of emotional content on pupil responses (e.g., Bradley et al., 2008; Hess, 1965) have presented stimuli for a relatively long period of time (many seconds). Studies that have examined the rate of extraction of emotional information from complex pictures, such as those from the International Affective Picture System (IAPS: Lang, Bradley, & Cuthbert, 1997), show that emotional information can be extracted from much briefer presentations. For example, Codispoti, Mazzetti, and Bradley (2009) showed that emotional information can be extracted from images as brief as 25 ms for images that are not masked, with little difference in performance for images of a much greater duration (such as 6,000 ms). This was true for both subjective ratings of the stimuli and for physiological measures such as the skin conductance response, the facial corrugator muscle response, and some ERPs (late positive response). This suggests that even brief, but unmasked, emotional stimuli might be able to modulate pupil size.

These earlier studies of pupil response to emotional pictures also provide little information as to what the participant did with their eyes during the presentation of the stimulus. It seems possible that varying patterns of eye movements may have occurred during the presentation of different pictures. For example, the participant might make a smaller number of saccadic eye movements during the fear image. Such an effect may serve to produce greater dilation in the fear condition via differences in eye movements rather than by fear producing a response in the sympathetic nervous system. We therefore tested whether emotional modulation of the pupil response was possible under conditions where the stimulus was so brief that no saccades could be initiated. We used stimuli presented for 100 and 300 ms, which are, respectively, well below or around the normal latency of saccadic movement initiation (Gilchrist, 2011; Miles & Kawano, 1987; Sumner, 2011).

### Method

#### Participants

We recruited 22 university students (17 female) based on power calculations (G*Power; Faul, Erdfelder, Lang, & Buchner, 2007) using effect sizes determined from previous pupillometry studies that have explored emotion.^1^ Average age was 25.91 years. Participants were asked to not wear bifocal or varifocal glasses on the day of testing as well as being requested to not consume caffeine or smoke 60 min prior to testing as this can influence pupil activity (Erdem et al., 2015; Wilhelm, Stuiber, Luedtke, & Wilhelm, 2014). These instructions were identical across subsequent experiments. All participants gave written informed consent to participate in the experimental procedures. All procedures were passed by the Ethics Committee of the School of Psychology, Cardiff University.

#### Materials and design

Twenty images,^2^ 10 neutral and 10 “fear” images, were selected from the IAPS. Fear images were selected from the affective fear category identified by Barke, Stahl, and Kroener‐Herwig (2012).

In pilot experiments, we looked at various stimulus properties to examine their effects on the pupillary response. We found that variations in stimulus luminance, luminance contrast, and color were all important factors that influenced the pupil's response to these complex images irrespective of any emotional content. Therefore, we attempted to match all our images along these dimensions using a standard image processing package (Adobe Photoshop Elements 12.0). First, all chromatic information was removed by converting the image to grayscale. Second, the overall luminance of each image was adjusted to be of the same mean luminance. Finally, the contrast of the image (defined as the standard deviation of all pixel values; Moulden, Kingdom, & Gatley, 1990) was adjusted to be equal to the specified level. Hence, all images had equivalent mean luminance, mean contrast, and (lack of) color content.^3^

Each test stimulus was preceded by a blank gray screen presented for 2,000 ms, which was luminance‐matched to the target stimulus. This blank screen also included a centered isoluminant fixation cross for the first 1,000 ms. The same blank gray screen followed all target stimuli as a recovery slide and was presented for 5,000 ms to allow pupil size to return to baseline.

To investigate the role of presentation duration on pupil response, each stimulus was presented once at each of four durations: 100, 300, 1,000, and 3,000 ms. The order of the 80 trials was randomized for each participant.

#### Pupil data acquisition, cleaning, and reduction^4^

All experiments took place in a dimly lit, sound‐proofed room. Pictures were displayed on a 48.30 cm display monitor, and each participant was seated 57 cm from the screen. A Tobii X2‐60 Hz eye tracker recorded pupil data throughout each task, which allowed relatively free movement of the head during the task. The hardware consisted of an inconspicuous eye‐tracking device located below the computer monitor that captured eye movements by illuminating the pupil via an infrared light sources and using two image sensors to record the reflection patterns. During recording, the eye tracker collected data every 16.67 ms. All measurements in this paper refer to the diameter of the pupil and are expressed in millimeters. The eye tracker was calibrated for each participant before each task using a five‐point calibration screen.

Data were recorded throughout each trial. We interpreted any pupil diameter change of ± 0.38 mm within a 20‐ms interval as random fluctuations and removed these (Partala & Surakka, 2003). We also deleted data points surrounding missing data (within 33.34 ms) to avoid anomalous readings. A prestimulus baseline pupil size average of 200 ms (Leknes et al., 2013) was calculated for each trial and subtracted from each subsequent data recording to establish baseline‐corrected pupil response across the trial. We calculated the mean pupil response at every data time point (every 16.67 ms) across trials for each condition. Mean pupil response was not calculated at data time points where there were missing data for more than 50% of condition trials. Linear interpolation was used to estimate pupil diameter where missing pupil samples led to large fluctuations in the mean pupil change for the relevant condition, usually around image offset.

### Results

**Figure 1:**
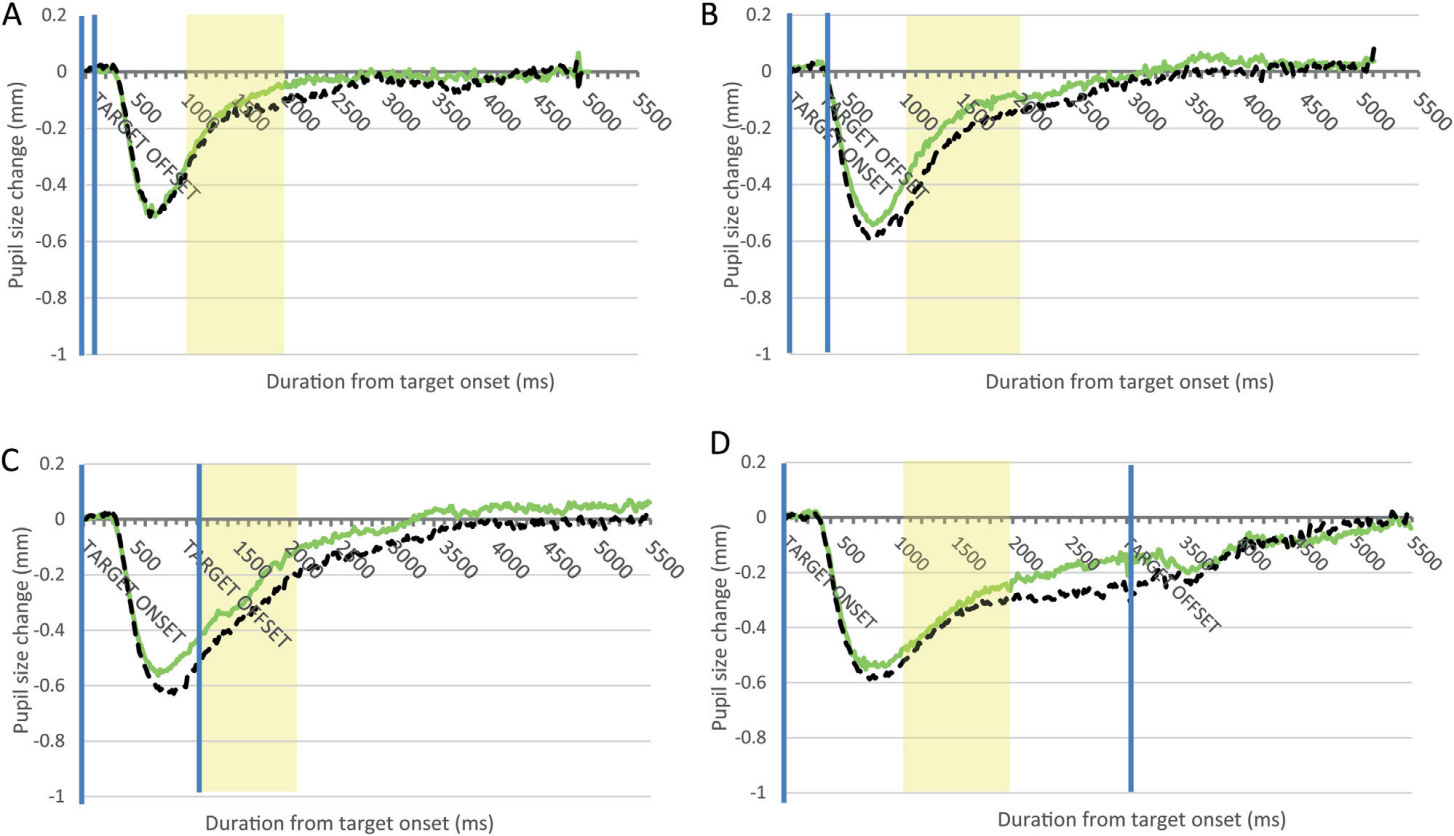
Data from Experiment 1. A: Pupil diameter is plotted as a function of the time since the target's onset across all participants. The black broken line is for the neutral stimuli, and the green continuous line is for the fear stimuli. The blue vertical lines mark the onset and offset of the target stimulus, while the yellow shaded region represents the time window that we used for statistical analysis of pupil size. The target was presented for 100 ms. B: Target duration was 300 ms. C: Target duration was 1,000 ms. D: Target duration was 3,000 ms.

Pupil diameter as a function of time from target image onset is shown in Figure 1. It appears that the pupil was larger in the fear stimuli trials than the control trials, and this occurred at all durations. It is also evident that pupil constriction is greater with increased presentation duration. To quantify each participant's affective pupil response, we calculated the average baseline‐corrected pupil size for the window 1,000–2,000 ms poststimulus onset for both fear and neutral stimuli. This time window was chosen as it avoids the initial pupil constriction that occurs in response to visual stimulation (reaching maximum constriction at approximately 800 ms). These data are displayed in Table 1. For the current experiment, 14.90% of total data was missing during our analysis response window. Average baseline‐corrected data were entered into a 2 × 4 analysis of variance (ANOVA) with factors of emotional content (fear vs. neutral) and duration (100, 300, 1,000, 3,000 ms). We obtained a main effect of stimulus emotional content, *F*(1,21) = 17.30, *p <* .001, η_p_^2^ = .45, a main effect of stimulus duration using Greenhouse‐Geisser correction, *F*(1.41,29.55) = 17.91, *p* < .001, η_p_^2^ = .46, but no significant interaction between emotion and duration, *F*(3,63) = 0.86, *p* = *n.s*., η_p_^2^ = .04.

**Table 1:** Mean Baseline‐Corrected Fear and Neutral Pupil Response Averaged Over 1,000–2,000 ms Postimage Onset

Target duration (ms)	100	300	1,000	3,000
Fear	−0.14 (0.03)	−0.18 (0.02)	−0.30 (0.05)	−0.35 (0.05)
Neutral	−0.18 (0.02)	−0.26 (0.03)	−0.38 (0.04)	−0.38 (0.05)
Difference	0.04	0.08	0.08	0.03
*p* value	.06	<.05	<.05	.12

*Note*. Standard errors are in parentheses.

The main effect of duration was further tested via post hoc comparisons. This showed that the pupils were larger (less constriction response) at 100 than at 300 ms, and at 300 than at 1,000 ms (*p*s < .01), but there was no significant difference between 1,000 and 3,000 ms.

## Experiment 2. The Pupil's Response to Emotions: Habituation

Anecdotal evidence suggests that many of the stimuli that we have used in the course of these experiments elicit a strong reaction when a person first sees them, but repeated presentation reduces this reaction. Habituation of response is a well‐studied phenomenon in psychology and physiology (Thompson & Spencer, 1966), and it seems possible that the modulation of the pupil to a particular emotion might reduce over repeated presentations. For example, responses in the amygdala to fear stimuli have been shown to reduce with repeated presentations (Bordi & Ledoux, 1992; Wilson & Rolls, 1993; Wright et al., 2001). Increasing the number of trials (either by repeating the same stimuli several times or by having a greater number of exemplars of the category) might therefore be expected to reduce the dilation of the pupil to the fear stimulus.

### Method

To investigate this issue, we performed an experiment using four blocks of 20 trials. In each block, we presented 10 neutral images and 10 fear images (the same stimuli used in Experiment 1) in a random order. Stimulus duration was 2,000 ms.

There was a gap of 3 min, consistent with Steiner and Barry (2014), between Blocks 2 and 3 to allow for a degree of pupil response recovery, where participants were asked to take a break but to remain seated in the same position. Blocks 1 to 2 and Blocks 3 to 4 ran consecutively with no gap. Twenty‐two participants (15 female) with an average age of 24.27 years took part in the experiment.

### Results

For the current experiment, 12.70% of total data was defined as missing during the analysis response window. However, in order to perform a 10 × 2 × 4 ANOVA with factors of trial position within‐block (1–10), emotional content (fear vs. neutral), and block (1–4), missing values were imputed with the mean value calculated using a Markov chain Monte Carlo multiple imputation method. Twenty imputations were calculated (Graham, Olchowski, & Gilreath, 2007). The ANOVA showed that the pupil was more dilated during the fear images than during the neutral images, *F*(1,21) = 23.79, *p* < .001, η_p_^2^ = .53, and the pupil was larger (or constricted less) across blocks, *F*(3,63) = 4.69, *p <* .05, η_p_^2^ = .18, but there was no main effect of position within block, *F*(4.72,99.05) = 0.67, *p* = *n.s*., η_p_^2^ = .03.

**Figure 2:**
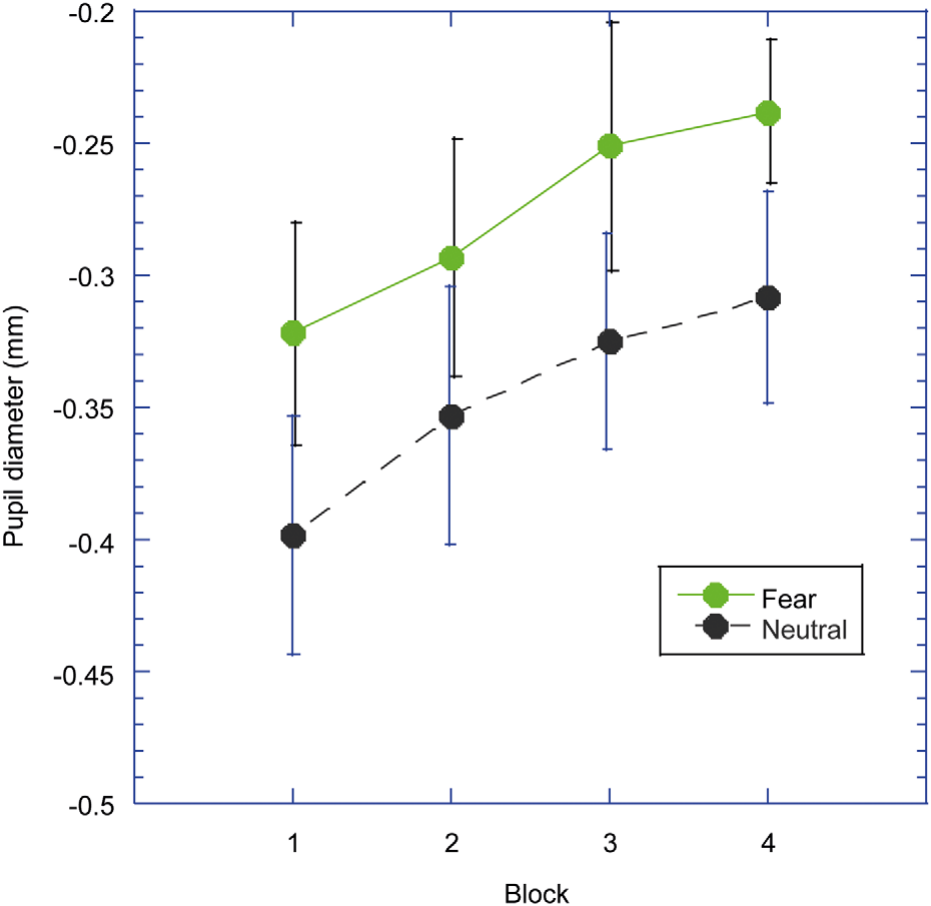
Data from Experiment 2. Mean pupil diameter is plotted for each of the blocks of trials. The black broken line is for the neutral stimuli and the green continuous line is for the fear stimuli. The error bars represent ± 1 standard error of the mean (*SEM*).

Our main hypothesis was that there might be a reduction in the size of the fear modulation effect such that large effects are produced in the early blocks, but small or nonexistent effects in later blocks. However, the interaction between block and the emotional content was not significant, *F*(3,63) = .08, *p* = *n.s*., η_p_^2^ = .00, and is illustrated in Figure 2. Further, there was no sign of any habituation of this effect within a block (two‐way interaction between trial and emotion, *F*(9,189) = 1.28, *p* = *n.s*., η_p_^2^ = .06. For completeness, the two‐way interaction between trial and block was not significant, *F*(27,567) = 1.40, *p* = *n.s*., η_p_^2^ = .06, nor was the three‐way interaction between trial, emotional content, and block, *F*(27,567) = 1.14, *p* = *n.s*., η_p_^2^ = .05.

## Experiment 3. The Pupil's Response to Emotions: Passive Versus Active Viewing

In most experiments that examine the pupil's reaction to emotional stimuli, the participant has been asked to simply view the picture passively. However, in most behavioral paradigms, the participant is required to perform some sort of judgment about the stimuli, such as the emotion portrayed within the image (e. g., Copestake, Gray, & Snowden, 2013). In the latter sort of paradigm, the participant is directly attempting to attend to the emotional elements of the image, and one might assume that such active viewing might magnify the effect of the emotional content of the stimuli. Indeed, there are many experiments that show that attention to emotion has a stronger behavioral effect, such as in experiments on affective priming (Gawronski, Cunningham, LeBel, & Deutsch, 2010) and on responses within some areas of the brain, such as the amygdala (Anderson, Christoff, Panitz, De Rosa, & Gabrieli, 2003), which are heavily implicated in emotional processing.

While there appears to be no previous study investigating the effects of passive versus active viewing on the emotional modulation of the pupillary light response, there is a large literature on the effects of cognitive load on pupil size. Just and Carpenter (1993) have described the pupillary response as a reflection of the amount of resources allocated to the task: the greater the task difficulty, the larger the pupillary dilation (Prehn, Heekeren, & van der Meer, 2011; van der Meer et al., 2010). During difficult tasks that involve high cognitive load, the pupil diameter increases even in the baseline period, presumably representing anticipatory resource allocation (Steinhauer et al., 2004).

In Experiment 3, we manipulated the viewing conditions. In one condition, termed passive viewing, the participants were asked simply to view the pictures—thus mimicking the conditions of Experiments 1 and 2. In the second condition, termed active viewing, we instructed the participant to consider whether an emotion was being portrayed by the picture and to signal this via a button response when the picture was removed.

### Method

Twenty‐two female participants were recruited with an average age of 20.10 years old. Stimulus selection was identical to Experiments 1 and 2 with a stimulus presentation time of 2,000 ms. The experiment was between‐subjects, with 11 participants completing the passive condition and 11 participants taking part in the active condition. In the active condition, participants were asked to view each image and then make a decision whether that image was emotive or neutral, whereas participants in the passive condition were asked simply to view the image.

### Results

**Figure 3:**
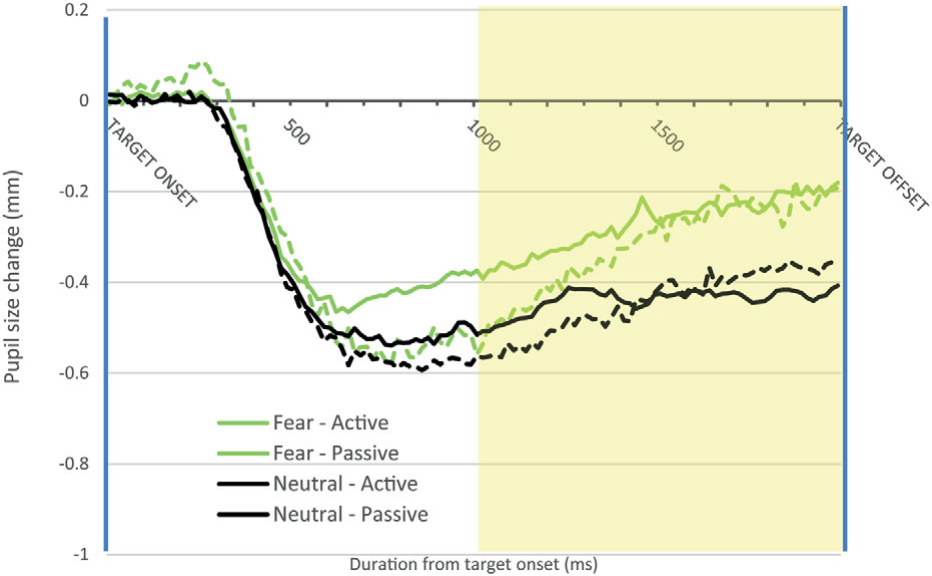
Data from Experiment 3. Pupil diameter is plotted as a function of the time since target onset across all participants. The black lines are for the neutral stimuli and the green lines for fear stimuli. The broken lines are for the passive viewing conditions and the continuous lines are the active viewing conditions. Other conventions are as in Figure 1.

Baseline‐corrected data are displayed in Figure 3. For the current experiment, 14.60% of total data was defined as missing during the analysis response window. These graphs have been terminated at 2,000 ms poststimulus onset as, at this time, the participants in the two conditions performed very different physical tasks (button press or passive view), and so comparisons are not meaningful.

The effect of emotional content of the target image appears to occur in both conditions with approximately the same magnitude. A 2 × 2 ANOVA, with factors of emotional content (neutral and fear) and viewing mode (passive and active), confirmed these observations. There was a main effect of emotional content, *F*(1,20) = 13.33, *p* < .05, η_p_^2^ = .40, but no main effect of viewing mode, *F*(1,20) = 0.04, *p* = *n.s*., η_p_^2^ = .00, and no interaction was found, *F*(1,20) = 0.29, *p* = *n.s*., η_p_^2^ = .01.

## General Discussion

Experiment 1 looked at the effect of duration of the test image. Crucially, we found no effect of image duration on the emotional modulation of the pupil, suggesting a comparable dilation due to emotional content at even the briefest stimulus duration we used. At these brief durations, no saccadic eye movements are possible. Hence, alternate explanations of the emotional modulation of the pupil based on differential eye movements when presented with emotional stimuli are not feasible. Given that the emotional effect occurs during such brief durations, it seems unnecessary to postulate that another mechanism produces the emotional effect at longer durations, although our data cannot rule out such a possibility.

Nuske et al. (2014) also varied the duration of their affective facial stimuli and measured pupil responses in children with autism and in typically developing children. Though the pattern of results is complex, they show evidence of emotional modulation of the pupil response for brief stimuli in the typically developing children.

Our findings appear commensurate with the findings of Codispoti, Bradley, and Lang (2001), who demonstrated that, for unmasked stimuli, both ratings of arousal and pleasure as well as psychophysiological measures such as skin conductance response were well developed by around 80 ms and did not increase with much longer stimulus presentations. However, we stress that, in our experiment, the test stimulus was not masked after presentation and, hence, visual processing of the scene might continue beyond this presentation period. Future experiments might seek to limit the duration of processing by introducing a “backward masking” procedure (where the target stimulus is immediately followed by a pattern mask that stops further visual processing of the target). It would also be of great interest to extend this experiment to even briefer intervals where the detection of the emotional content of the stimulus no longer produces a conscious or reportable sensation (so‐called subliminal presentations: see Nuske et al., 2014) given the claims that areas such as the amygdala not only can respond to subliminal stimuli, but may even give greater responses than to supraliminal stimuli (Williams et al., 2006)—though also see Hoffmann, Lipka, Mothes‐Lasch, Miltner, and Straube (2012). We should also note that we only analyzed the pupil response for a limited window of time shortly after stimulus onset (1,000–2,000 ms). It seems likely that, if a later or longer response window were used, longer duration stimuli would produce greater dilations than brief ones due to the continued presence of, for example, a threat stimulus.

Experiment 2 examined whether the emotional modulation of the pupil response would habituate across the course of a typical experiment. Though we found some evidence for a habituation of the pupillary light reflex overall, this was the same for both the fear and neutral stimuli. Hence, the effect due to the emotion elicited by the stimulus did not habituate across blocks, nor did we find any evidence for changes within blocks of trials. Our results seem commensurate with those reported by Bradley and Lang (2015). They presented participants with stimuli that were either novel or had been previously viewed. They show that, for all stimuli, irrespective of valence, there is a novelty effect such that the old items produce greater pupil dilation. Further, the effects were found whether the repetitions were massed (presenting the same stimulus on repeated trials) or distributed (with other stimuli intervening, as in the present experiments). Hence, both the present experiments and those of Bradley and Lang (2015) demonstrate that, while there is an increase in pupil dilation with repeated presentations (or more strictly speaking, a lesser constriction of the pupillary light reflect), this is not specific to the emotional stimuli, and the difference between emotional and nonemotional stimuli is preserved across presentations.

It is noteworthy that most psychophysiological measures of emotion processing are thought to habituate with repeated presentations. For example, Bradley, Lang, and Cuthbert (1993) show that the emotional modulation of skin conductance responses and facial corrugator muscle activity both decrease with repeated presentation of emotional stimuli. However, the same group shows that the emotional modulation of the startle response did not reduce with repeated presentations (even though the actual startle response itself habituated). Hence, it appears that some emotional measures do habituate while others do not, or at least the rate of habituation may vary for different psychophysiological components (Bradley, 2009). This lack of habituation of the emotional modulation of the pupil response has useful practical applications as it suggests that researchers can increase the number of trials in their experiments in order to improve the signal‐to‐noise ratio of the measurement without suffering from a loss of signal due to habituation.

In Experiment 3, we investigated if active processing of the emotional content of the images might increase the amount of emotional modulation in comparison to a passive viewing condition. We did not find any difference between these conditions. This result is consistent with the idea that the structures that give rise to the emotional modulation of the pupil may be automatic and beyond conscious control, a claim that has been made for some brain structures that may mediate the emotional modulation of pupil responses (e.g., Whalen et al., 1998), though this is disputed by others (Hoffmann et al., 2012). We stress, however, that we did not attempt a very stringent test of whether attention could ever modulate the effect of emotion on the pupil's response. Our aim was merely to demonstrate that the emotional effect generalizes from simple passive viewing to conditions where the person must try and process the emotional content of the image. We should also note that this experiment only tested female participants. Further experiments are needed to examine if the emotional stimuli continue to produce such changes in pupil size under conditions where attention to the emotional stimulus is withdrawn by, say, the processing of other competing stimuli (see De Cesarei, Codispoti, & Schupp, 2009) or in male participants.

## Footnotes

1 All subsequent experiments use similar power calculations.

2 IAPS images used were—neutral: 2190, 2745.1, 2850, 2870, 2036, 2214, 2383, 2514, 5731, 2393; fear: 6263, 6250, 1525, 6370, 6231, 1304, 1301, 6242, 9901, 5973.

3 This treatment was identical for all experiments reported.

4 Data acquisition, cleaning, and reduction procedures are identical for subsequent experiments.
